# Society of Homœopathicians

**Published:** 1895-07

**Authors:** 


### SOCIETY OF HOMŒOPATHICIANS.

The first annual meeting of the Society of Homœopathicians was successful in every particular. It was held at the Oriental Hotel, Manhattan Beach, June 26th and 27th.

The papers were interesting and the discussions most instructive. One thing was to be regretted, there was no stenographer present to report the proceedings, and, as is usually the case, the remarks and discussions were fully as important as the papers, if not more so.

This will be remedied at the next meeting, at which a stenographer will be present.

The two Bureaus of Homœopathic Philosophy and Clinical Medicine were well represented by papers of thought and experience. An especially able paper was that by Dr. Thomson, Chairman of the Bureau of Homœopathic Philosophy. It will be enjoyed by readers of THE HOMŒOPATHIC PHYSICIAN when it appears in its pages.

There was a unanimous opinion that the meeting was not long enough, and it was decided that next year three days would not be too much for the sessions of the society and for the social intercourse which is one of the most delightful parts of these yearly gatherings.

The society adjourned feeling that its first attempt was a great success, that a home was established for pure Homœopathy, and that next year the meeting would be even more enjoyable. K.

